# Proving Lipid Rafts Exist: Membrane Domains in the Prokaryote *Borrelia burgdorferi* Have the Same Properties as Eukaryotic Lipid Rafts

**DOI:** 10.1371/journal.ppat.1003353

**Published:** 2013-05-16

**Authors:** Timothy J. LaRocca, Priyadarshini Pathak, Salvatore Chiantia, Alvaro Toledo, John R. Silvius, Jorge L. Benach, Erwin London

**Affiliations:** 1 Department of Molecular Genetics and Microbiology, Stony Brook University, Stony Brook, New York, United States of America; 2 Department of Biochemistry and Cell Biology, Stony Brook University, Stony Brook, New York, United States of America; 3 Department of Biochemistry, McGill University, Montreal, Quebec, Canada; Medical College of Wisconsin, United States of America

## Abstract

Lipid rafts in eukaryotic cells are sphingolipid and cholesterol-rich, ordered membrane regions that have been postulated to play roles in many membrane functions, including infection. We previously demonstrated the existence of cholesterol-lipid-rich domains in membranes of the prokaryote, *B. burgdorferi*, the causative agent of Lyme disease [LaRocca *et al.* (2010) Cell Host & Microbe 8, 331–342]. Here, we show that these prokaryote membrane domains have the hallmarks of eukaryotic lipid rafts, despite lacking sphingolipids. Substitution experiments replacing cholesterol lipids with a set of sterols, ranging from strongly raft-promoting to raft-inhibiting when mixed with eukaryotic sphingolipids, showed that sterols that can support ordered domain formation are both *necessary* and *sufficient* for formation of *B. burgdorferi* membrane domains that can be detected by transmission electron microscopy or in *living* organisms by Förster resonance energy transfer (FRET). Raft-supporting sterols were also necessary and sufficient for formation of high amounts of detergent resistant membranes from *B. burgdorferi*. Furthermore, having saturated acyl chains was required for a biotinylated lipid to associate with the cholesterol-lipid-rich domains in *B. burgdorferi*, another characteristic identical to that of eukaryotic lipid rafts. Sterols supporting ordered domain formation were also necessary and sufficient to maintain *B. burgdorferi* membrane integrity, and thus critical to the life of the organism. These findings provide compelling evidence for the existence of lipid rafts and show that the same principles of lipid raft formation apply to prokaryotes and eukaryotes despite marked differences in their lipid compositions.

## Author Summary

Specialized domains (“lipid rafts”) rich in specific membrane lipids (sphingolipids and cholesterol) have been proposed to form in the cell membranes of higher organisms, and to be of functional importance. We recently found that domains can be detected in the membranes of the bacterium that causes Lyme disease, *Borrelia burgdorferi*. In this report it is shown that, despite a lack of sphingolipids in *B. burgdorferi*, these domains have all the characteristic properties of lipid rafts, and can be detected in living *B. burgdorferi*. This shows that true lipid rafts can form in bacteria. In addition, it is shown that sterols having a structure that promotes lipid raft formation are necessary and sufficient for those sterols to maintain *B. burgdorferi* membrane integrity. This is suggestive of a role for membrane domains in *B. burgdorferi* membrane integrity. Therefore, interfering with lipid raft formation may have biomedical applications in combatting *B. burgdorferi* infections.

## Introduction

The spirochete *Borrelia burgdorferi* is the causative agent of Lyme disease [1], [2], a tick-borne illness that can have manifestations in the skin, heart, joints, and nervous system of mammals [3]. *B. burgdorferi* has outer and inner membranes, and the periplasmic space between these membranes contains flagellar bundles. The flagella contribute to *B. burgdorferi* morphology [4], and are not exposed to the extracellular environment unless the outer membrane is damaged [3], [5].

*B. burgdorferi* membranes contain phosphatidylcholine, phosphatidylglycerol and lipoproteins [6]–[8]. They also contain free cholesterol, two cholesterol glycolipids (acylated cholesteryl galactoside (ACGal) and cholesteryl galactoside (CGal)), and the glycolipid monogalactosyl diacylglycerol (MGalD) [9]–[12]. Only a few other bacteria are known to incorporate cholesterol into their membranes [13]–[17].

In eukaryotic cells, sterols (together with sphingolipids having saturated acyl chains) are believed to participate in the formation of ordered membrane domains called rafts, which co-exist with disordered membrane domains, and which are thought to play an important role in many membrane functions [18]–[23]. In model membranes, ordered sterol-rich domains are readily detected [24]. However, it has been difficult to characterize rafts in eukaryotic cells due to their small size and dynamic properties, and their existence remains controversial.

We previously presented evidence that lipid microdomains containing cholesterol glycolipids exist in *B. burgdorferi* membranes [25]. In this study we demonstrate that the formation of these domains have all the hallmarks of lipid rafts and that the domains are present in living *B. burgdorferi*.

## Results

### Sterols with the ability to form ordered membrane domains are necessary and sufficient to form *B. burgdorferi* membrane domains that can be observed by Transmission Electron Microscopy (TEM)

The hypothesis that *B. burgdorferi* domains are lipid rafts predicts that their formation should require lipids having the ability to form tightly packed domains. In previous studies we demonstrated that different sterols have a structure-dependent range of abilities to support formation of ordered raft lipid domains in model membrane vesicles [26]–[29]. Therefore, sterol substitution experiments were carried out in *B. burgdorferi* using sterols (Table S1 in Text S1) ranging from those that are strongly ordered domain forming to those that are ordered domain inhibiting [26]–[29].

Free cholesterol and cholesterol glycolipids from *B. burgdorferi* can be substantially removed from cells with methyl-β-cyclodextrin (MβCD) while phospholipids and MGalD are unaffected [25]. When depletion is followed by incubation of the spirochetes with a diverse set of sterols, thin layer chromatography (TLC) analysis of *B. burgdorferi* lipid extracts indicated that sterol substitution had taken place (Fig. S1 in Text S1). Sterol substitution was confirmed by a strong correlation between the ability of a sterol to support ordered domain formation in model membranes [26]–[29] and membrane order in *B. burgdorferi* membranes (in intact cells), as measured by the anisotropy of trimethylaminodiphenylhexatriene (TMADPH) fluorescence, subsequent to sterol substitution (Table S1 and Fig. S2 in Text S1).

**Figure 1:**
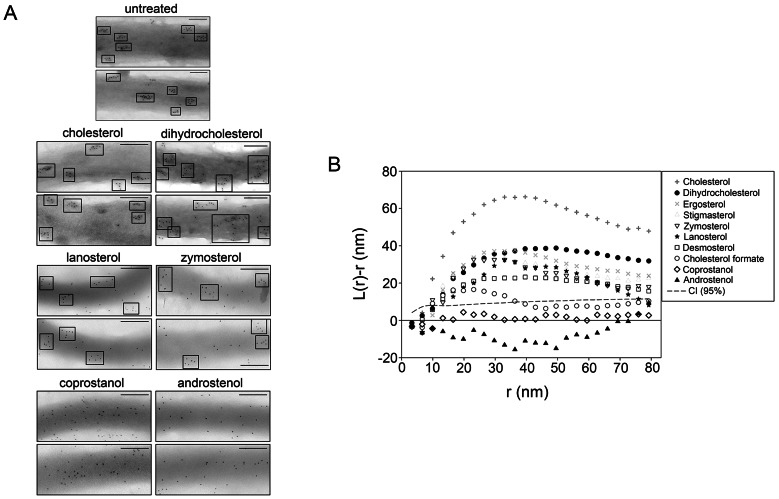
Domain formation in *B.* *burgdorferi* as determined by immunogold TEM analysis requires raft-supporting sterols. **A.** Representative negative-stain TEM images of *B. burgdorferi* substituted with the indicated sterols and probed for sterol glycolipids using a rabbit antibody to asialo-GM1 followed by a secondary anti-rabbit antibody conjugated to 6 nm colloidal gold. Micrographs show electron dense regions, which are portions of *B. burgdorferi* and show associated gold particles. Two images (of about 400 nm long segments) are shown for each sterol. Top row, sterols strongly supporting ordered domain formation; middle row, sterols with an intermediate ability to form ordered domains; bottom row, sterols that inhibit ordered domain formation [22], [26]–[28]. Boxes highlight sterol glycolipid clusters. Bars = 100 nm. TEM micrographs for additional sterols are shown in Fig. S3 in Text S1. **B.** Pooled “K-function” analysis of TEM experiments. The spatial distribution of gold particles is presented as curves representing the mean values of *L(r)-r* from images of three different bacteria from three independent sterol substitution experiments. Values of *L(r)-r* above the CI (95% confidence level, dashed line) indicate clustering (i.e. domain formation) of the sterol glycolipid at that specific length scale.

After sterol substitution, *B. burgdorferi* were prepared for immunogold negative stain TEM analysis to determine the effect of substitution upon cholesterol glycolipid-containing membrane microdomain formation (Fig. 1, Fig. S3 in Text S1). For this, TEM grids were probed with a rabbit antibody to asialo GM1, as this antibody cross-reacts with the cholesterol glycolipids of *B. burgdorferi* [25], [30], [31]. The ultrastructural appearance of the membrane microdomains in spirochetes whose native cholesterol was substituted with sterols that strongly promote lipid raft formation (ergosterol, cholesterol, dihydrocholesterol, and stigmasterol) was indistinguishable from the appearance of those in normal, untreated *B. burgdorferi* (Fig. 1A and Fig. S3 in Text S1) [25]. Distinct clusters were observed with a diameter of ∼50 nm, similar to those seen in *B. burgdorferi* prior to sterol substitution [25]. This is consistent with the raft-promoting behavior of these sterols in model membranes [26], [27].

Substitution of native cholesterol with sterols with intermediate raft-forming ability (desmosterol, lanosterol, zymosterol and cholesterol formate) also resulted in visually apparent glycolipid microdomains (Fig. 1A and Fig. S3 in Text S1). However, in the case of lanosterol and zymosterol unclustered sterol glycolipid molecules could also be seen (Fig. 1A).

Decreased microdomain organization was observed in cholesterol formate substituted spirochetes (Fig. S3 in Text S1), and was lost when *B. burgdorferi* were substituted with coprostanol or androstenol (Fig. 1A), sterols that inhibit raft formation.

The clustering of gold particles in the micrographs was also analyzed using Ripley's K-function (Fig. 1B and Fig. S4 in Text S1) [32]. The clustering parameter L(r)-r is a measure of the excess of particles, relative to that expected if the distribution is random within a region radius r around each particle. Values significantly higher than zero indicate clustering, while a value of zero indicates random particle distribution within the distance r around each particle. The results were in agreement with visual assessment of ultrastructure, with clustering (L(r)-r) values above the 95% confidence limit for all sterols with the exception of coprostanol and androstenol, which showed no evidence of clustering, and cholesterol formate, which showed very weak clustering values near the confidence index. The y-axis values peak at points corresponding to domain radii close to 20–40 nm. We conclude that a sterol having the ability to form ordered domains is both necessary and sufficient for that sterol to induce TEM-detected domain formation in *B. burgdorferi*.

### Sterols with the ability to form ordered membrane domains are necessary and sufficient for FRET-detected membrane domain formation in live *B. burgdorferi*

**Figure 2:**
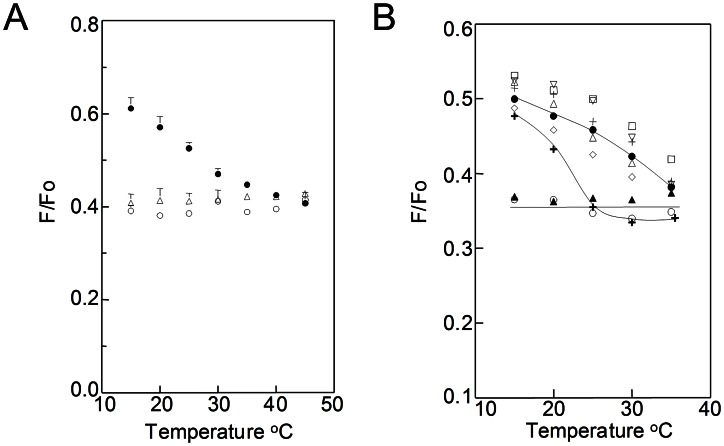
FRET detection of ordered domain formation as a function of temperature in *B.* *burgdorferi*. **A.** Demonstration of FRET assay performance in model membranes. Samples of small unilamellar vesicles contained 100 µM lipid and (in Fo samples) TMADPH or (in F samples) both TMADPH and ODRB. Vesicles were composed of 2∶1 (mol∶mol) POPC/chol (open circles), 2∶1 DOPC/chol (open triangles), or 1∶1∶1 DPPC/DOPC/chol (filled circles). F/Fo is the fraction of donor fluorescence unquenched by FRET. Average of duplicates and range are shown. **B.** Detection of ordered domain formation in *B. burgdorferi* by FRET. F and Fo samples contained *B. burgdorferi* (4×10^8^ cells/ml) with TMADPH (Fo samples) or both TMADPH and ODRB (F samples). Symbols: untreated cells (diamonds), cholesterol depleted cells (bold plus sign), or cells substituted with cholesterol (filled circles), dihydrocholesterol (open triangles), ergosterol (inverted, vertex down, triangles), lanosterol (plus sign), zymosterol (squares), coprostanol (filled triangles) or androstenol (open circles). Mean F/Fo values from four samples, or two in the case of untreated cells and lanosterol, are shown. For clarity, error bars are omitted in B. (Summary FRET data with error bars is shown in Figure S5 in Text S1.)

To rule out the possibility that membrane domains are induced by the fixation used to prepare specimens for immunogold TEM analysis, a fluorescence resonance energy transfer (FRET) method was developed to detect domains in living cells. In this method, weak FRET is observed when co-existing ordered and disordered lipid domains are present because the donor, TMADPH, partitions moderately into ordered domains and so partially segregates from the acceptor, octadecylrhodamine B (ODRB) which partitions preferentially into disordered lipid domains [33], [34]. The FRET method was calibrated in model membranes (Fig. 2A). At low temperatures, there was higher TMADPH fluorescence (higher F/Fo = weaker FRET) in vesicles having a composition (dipalmitoylphosphatidylcholine (DPPC)/dioleoylphosphatidylcholine (DOPC)/cholesterol) in which segregation into DPPC-rich ordered and DOPC-rich disordered domains occurs [35] than in vesicles (DOPC/cholesterol or palmitoyloleoylphosphatidylcholine (POPC)/cholesterol) forming a homogeneous bilayer. At higher temperatures, in DPPC/DOPC/cholesterol samples lipid segregation is lost due to melting of the ordered domains, and FRET levels come close to that in homogeneous vesicles, which exhibit temperature-independent FRET.

When TMADPH and ODRB were added to living *B. burgdorferi* cells, weak FRET which increases at higher temperatures is observed (Fig. 2B and Fig. S5 in Text S1). This indicates that there is formation of co-existing ordered and disordered domains up to at least ∼35–40°C. The FRET experiments were repeated after sterol substitutions (Fig. 2B). Weak FRET which increased at higher temperatures was observed with sterols that strongly or moderately support ordered domain formation. In contrast, strong, temperature-independent FRET was observed after substitution with androstenol and coprostanol, sterols that do not support ordered domain formation, and for which domains were not observed in TEM. Thus, FRET-detected domain formation in living cells has the same dependence upon sterol structure as does TEM-detected domain formation, confirming that the latter is not an artifact of fixation. Partial depletion of cholesterol lipids with MβCD without subsequent sterol substitution did not totally abolish domain formation at lower temperatures, but did decrease their thermal stability, as shown by the transition from weak to strong FRET occurring at a lower temperature (Fig. 2B). This indicates that residual cholesterol lipids remaining after extraction are sufficient to form some ordered microdomains.

### Sterols with the ability to form ordered membrane domains are necessary and sufficient for isolation of detergent resistant membranes from *B. burgdorferi*

The ability to form ordered domains was evaluated next by isolation of detergent resistant membranes (DRM) from sterol-substituted *B. burgdorferi* cells. We previously showed that DRM containing cholesterol glycolipids, and the lipid-anchored proteins outer surface protein A (OspA) and outer surface protein B (OspB), can be isolated from *B. burgdorferi* [25]. Resistance to solubilization by detergent is a characteristic property of ordered state domains in membranes [36], [37]. Disordered lipid domains dissolve in detergents such as TX-100 while cholesterol-rich ordered domains do not. Although it has been claimed that the detergent TX-100 can induce domain segregation under some conditions, we recently found that this does not occur, and instead the effect of detergent is to coalesce pre-existing ordered domains into larger domains [38].

**Figure 3:**
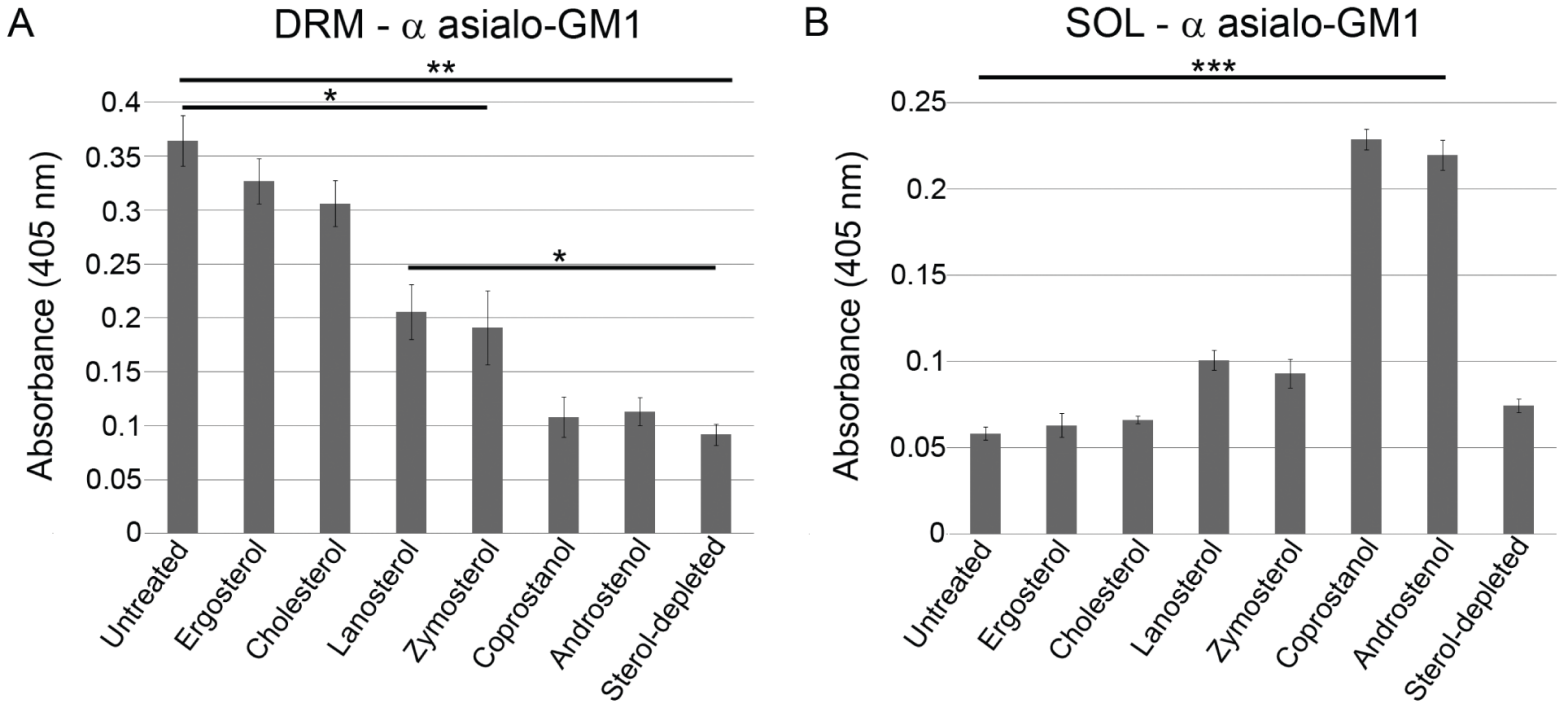
Sterol-dependence of ordered domain formation in *B.* *burgdorferi* as determined by detergent (TX-100) resistance. Levels of cholesterol glycolipids in: **A.** DRM fractions, or **B.** soluble (SOL) fractions based on ELISA assay. Mean values and standard error of the mean from three experiments are shown. One-way ANOVA, *** P<0.001, ** P<0.01.

Ergosterol and cholesterol gave the highest yield of cholesterol glycolipids in DRM while androstenol and coprostanol, which inhibit domain formation, gave the lowest yield of DRM (Fig. 3A). Lanosterol and zymosterol, with an intermediate ability to support ordered domain formation gave intermediate DRM levels. The levels of cholesterol glycolipids in the TX-100-soluble fractions showed an inverse pattern (Fig. 3B). An analogous pattern for the sterol structure-dependence of DRM association was found for OspA and OspB levels in DRM and soluble fractions (Fig. S6 in Text S1).

It should be noted that although DRM are prepared at 4°C, as is conventional [36], DRM containing cholesterol glycolipids, plus both OspA and Osp B, could also be isolated from *B. burgdorferi* at 33°C (Fig. S7 in Text S1). Because *B. burgdorferi* is exposed to a wide range of ambient temperatures in ticks and mammals, both 4°C and 33°C are physiologically relevant.

### Partitioning of biotinylated lipid probes shows *B. burgdorferi* cholesterol glycolipid domains preferentially accumulate saturated acyl chain lipids

**Figure 4:**
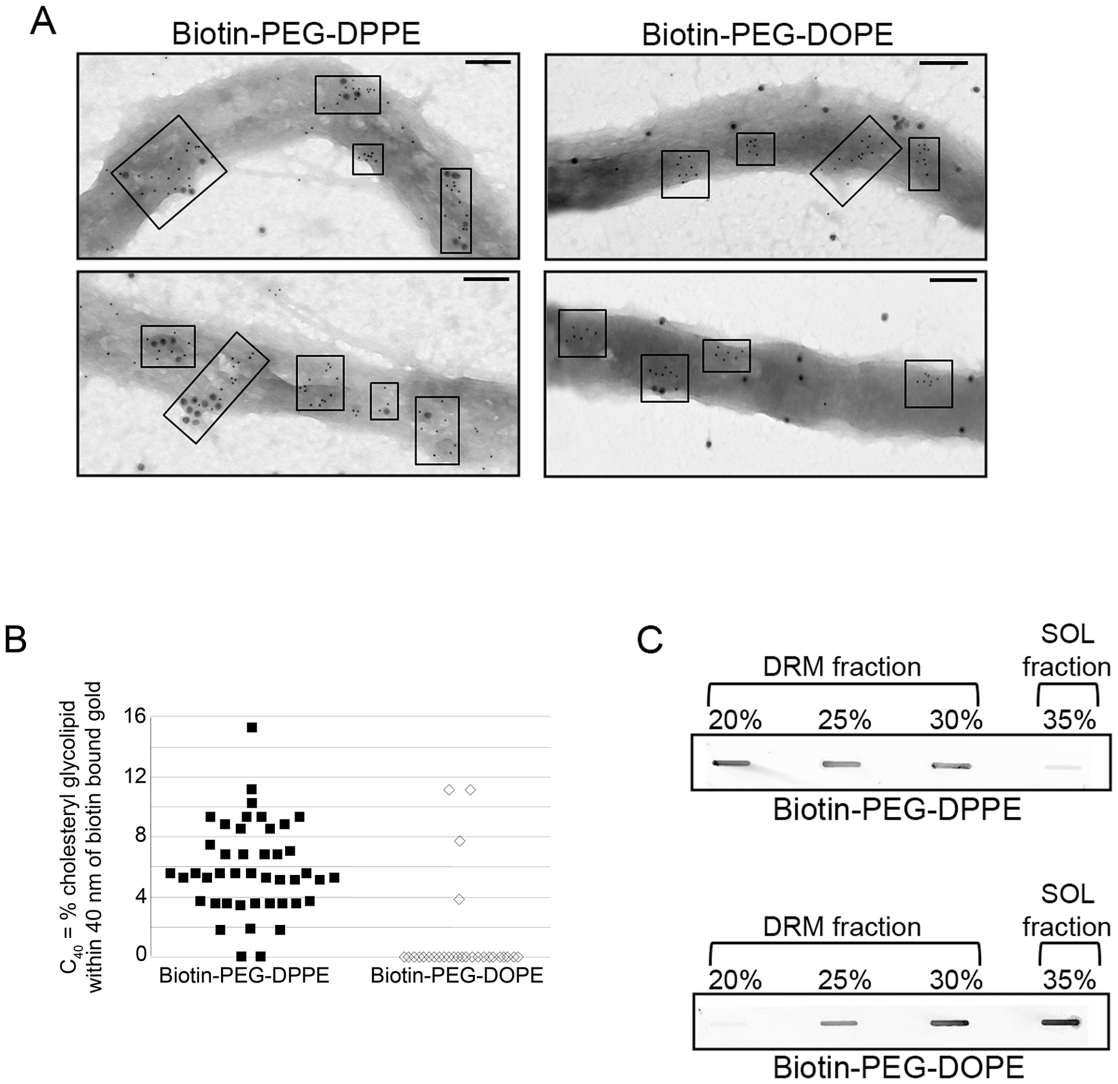
Only lipids associating with ordered domains colocalize with *B.* *burgdorferi* sterol glycolipid domains. **A.** Representative negative-stain TEM images showing localization of biotin-PEG-DPPE (left) and biotin-PEG-DOPE (right) as detected by anti-biotin and second antibody conjugated to 15 nm gold particles, in relation to cholesterol glycolipids, detected by antibody conjugated to 6 nm gold particles as in Figure 1. Electron dense areas in micrographs are sections of bacteria. Boxes denote clusters of sterol glycolipids. Notice the difference in large gold particle proximity to small gold particle clusters for biotin-PEG-DOPE and biotin-PEG-DPPE. Bars = 100 nm. **B.** Quantification of biotinylated lipid co-localization with cholesterol glycolipids. The co-localization parameter C_40_ (see Materials and Methods) indicates the percent of 6 nm gold particles within 40 nm of a 15 nm gold particle. C_40_ was calculated for both biotin-PEG-DOPE and biotin-PEG-DPPE samples from the mean of three different images. **C.** Representative immunoblots of *B. burgdorferi* fractions following TX-100 treatment and density gradient separation probed with anti-biotin antibodies.

Lipids having only saturated (e.g. palmitoyl) acyl chains tend to associate strongly with ordered lipid domains while lipids with acyl chains having cis double bonds (e.g. oleoyl), which act as kinks, pack poorly into, and so do not associate well with, ordered domains [19], [39]. To determine if *B. burgdorferi* membrane domains specifically associate with lipids bearing saturated acyl chains, their association with biotin-polyethyleneglycol-dipalmitoylphosphatidylethanolamine (biotin-PEG-DPPE) and biotin-polyethyleneglycol-dioleoylphosphatidylethanolamine (biotin-PEG-DOPE) [40] was compared (Fig. 4). Previous studies showed that biotin-PEG-DPPE has a significant affinity for ordered membrane domains while biotin-PEG-DOPE partitions more strongly into disordered domains [40].

The association of the biotin lipids to the cholesterol glycolipid domains was evaluated by negative-stain immunogold TEM using antibodies to biotin. Biotin-PEG-DPPE colocalized with or adjacent to cholesterol glycolipid domains (Fig. 4A, left), while biotin-PEG-DOPE did not (Fig. 4A, right). Analysis of the distances between biotinylated and cholesterol glycolipids confirmed visual results (Fig. 4B). This behavior confirms that the cholesterol glycolipid domains seen by TEM contain lipids in an ordered state.

The association of biotin-PEG lipids with *B. burgdorferi* DRM was also measured. DRM and soluble fractions were probed with anti-biotin antibodies. Biotin-PEG-DPPE was found to partition primarily into *B. burgdorferi* DRM fractions while most of the biotin-PEG-DOPE partitioned into the soluble fraction (Fig. 4C). These results indicate that *B. burgdorferi* DRM also have properties of ordered lipid domains.

### Sterols with the ability to form ordered membrane domains are necessary and sufficient to maintain normal *B. burgdorferi* cell morphology

**Figure 5:**
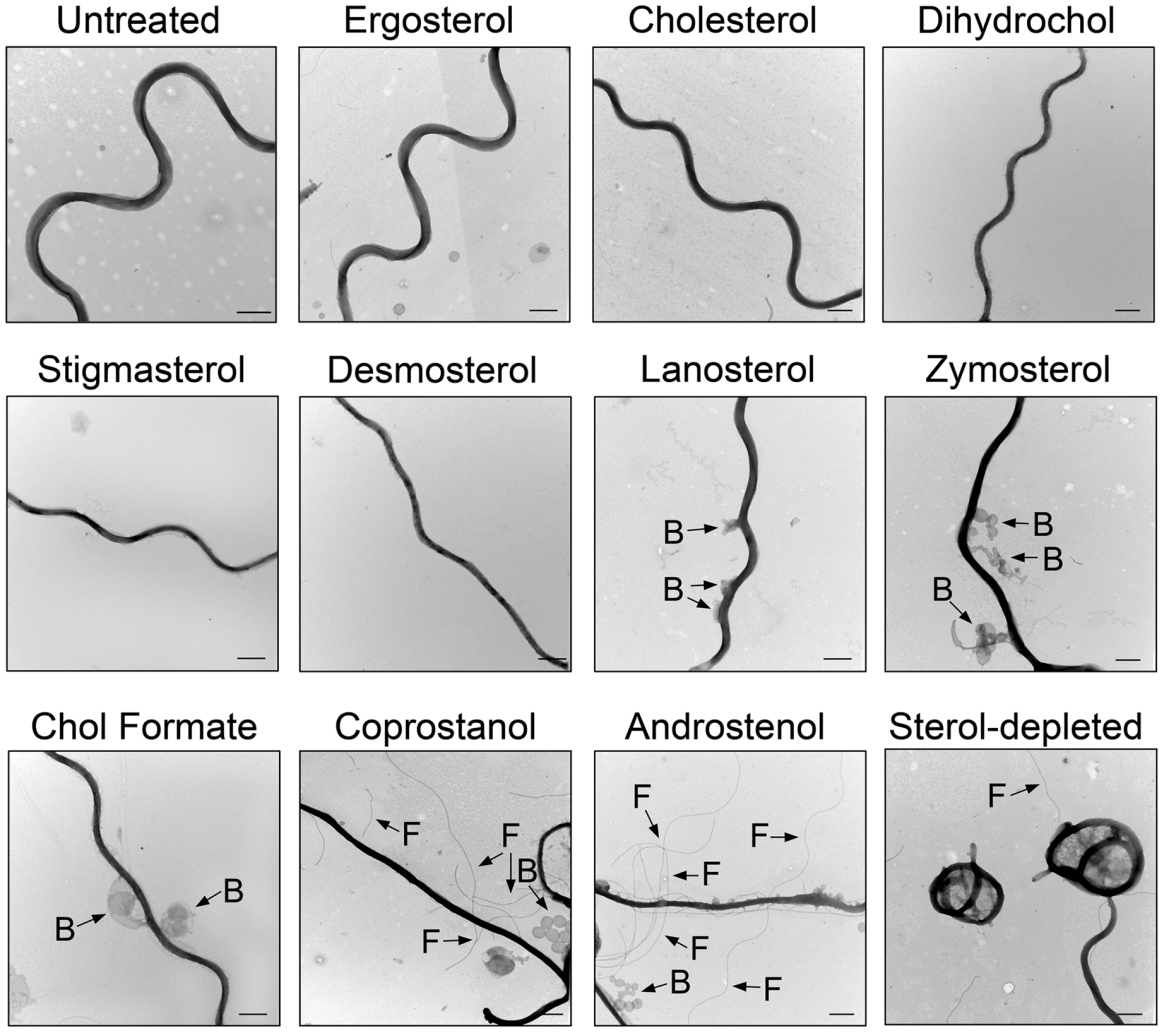
Effect of sterol substitution on the morphology of *B.* *burgdorferi*. Representative negative-stain TEM images of untreated *B. burgdorferi* or *B. burgdorferi* incubated 5 h at 33°C after substitution with the indicated sterols. B = Attached membrane vesicles (“blebs”). F = .flagella. Bars = 500 nm.

To test whether sterol substitution can affect other membrane properties, sterol-substituted *B. burgdorferi* were analyzed 5 h after sterol substitution by negative-stain TEM to assess sterol-dependent changes in spirochete morphology (Fig. 5). When substituted with strongly raft-forming sterols, *B. burgdorferi* cells showed normal planar wave morphology with a smooth surface and no release of periplasmic flagella (Fig. 5). Spirochetes substituted with sterols having an intermediate ability to support raft formation showed modest loss of planar wave morphology and with some of these sterols (lanosterol, zymosterol and cholesterol formate) membrane vesicles were observed protruding from the cells (Fig. 5). Spirochetes substituted with sterols that inhibit ordered domain formation completely lost their planar wave shape, forming straight cells or occasionally (not shown) a tightly curled morphology. They also exhibited greater formation and release of membrane vesicles, and exposure/release of periplasmic flagella into the external environment, suggesting loss of outer membrane integrity (Fig. 5). Spirochetes treated with MβCD but without sterol substitution coiled and showed a lesser release of periplasmic flagella after 5 h (Fig. 5). These experiments show that sterols having raft-forming properties contribute to the characteristic morphology of *B. burgdorferi*.

### The raft-forming ability of sterols correlates with effects on *B. burgdorferi* membrane permeability and integrity

**Figure 6:**
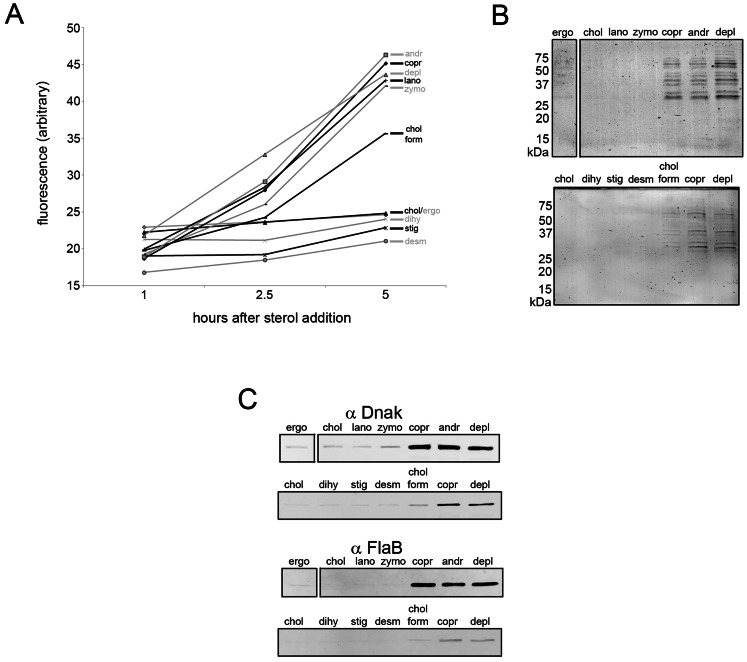
Sterols that differ in raft-forming ability have differential effects on *B.* *burgdorferi* membrane permeability. **A.** Propidium iodide staining of *B. burgdorferi* substituted with the indicated sterols. The x axis shows incubation time at 33°C after sterol substitution. The mean of three experiments each of which had three samples is shown. Standard error of the mean values (not shown to enhance figure clarity) were typically ±1 fluorescence units and did not exceed ±2 fluorescence units for any data point. **B.** Representative Coomassie-stained SDS-PAGE gels of supernatants from *B. burgdorferi* 5 h after substitution with the indicated sterols. Approximate M.W. shown at left of gels. **C.** Representative immunoblots showing the release of the cytosolic chaperone DnaK and periplasmic flagella subunit FlaB into the supernatants 5 h after substitution with the indicated sterols. Key; depl = sterol-depleted, ergo = ergosterol, chol = cholesterol, lano = lanosterol, zymo = zymosterol, copr = coprostanol, andr = androstenol, chol form = cholesterol formate, stig = stigmasterol, dihy = dihydrocholesterol, desm = desmosterol.

To evaluate membrane integrity in more detail, the effect of sterol substitutions on *B. burgdorferi* membrane permeability was evaluated using propidium iodide. When membrane permeability increases or membrane integrity is lost, propidium iodide binds to DNA and becomes fluorescent. Substitution with sterols that strongly promote raft formation, as well as desmosterol, did not produce an increase in incorporation of propidium iodide by *B. burgdorferi* cells over a 5 h time period (Fig. 6A). Other than desmosterol, sterols with an intermediate or inhibitory effect on raft formation caused a nearly linear increase in propidium iodide staining between 1 h and 5 h, as did sterol-depletion (Fig. 6A), indicating increased membrane permeability.

As propidium iodide staining does not distinguish between an increase in membrane permeability and a total loss of membrane integrity (which would lead to spirochete death), *B. burgdorferi* substituted with different sterols were evaluated for protein release 5 h after sterol substitution. Protein release (including that of cytoplasmic chaperone DnaK and FlaB (from periplasmic flagella) was not detected after substitution with strongly or moderately raft-forming sterols while the converse was true for raft-inhibiting sterols (Fig. 6B and C). Thus, sterols with a strong raft-forming ability are necessary to maintain fully normal membrane impermeability, while either a strong or moderate raft-forming ability are necessary and sufficient to prevent total loss of membrane integrity and spirochete death (see Discussion). This loss of membrane integrity appears to involve osmotic lysis (data not shown) as protein release was prevented in the presence of an osmoprotectant dextran500 [41].

## Discussion

### *B. burgdorferi* membrane domains are true lipid rafts

Our prior study showed membrane domains existed in fixed *B. burgdorferi* cells, and that cholesterol lipids played a role in their formation, suggesting they would be analogous to the lipid rafts proposed to form in eukaryotic cells. However, we did not show that these domains shared the characteristic properties of eukaryotic lipid rafts, or that they formed in living *B. burgdorferi*.

The studies in this report show that *B. burgdorferi* membrane domains are true lipid rafts. Having raft-forming properties (based on prior studies with eukaryotic sphingolipids [26]–[29]) was both *necessary* and *sufficient* for sterols to support the formation of *B. burgdorferi* membrane domains as judged by TEM, detergent-resistance, and FRET. The observation of ordered domain formation by FRET was especially important as it indicates that domains form in live *B. burgdorferi*. In addition, membrane domains selectively incorporated molecules membrane-anchored via saturated acyl chains, but not analogous molecules having unsaturated acyl chains, the other key characteristic property of eukaryotic ordered lipid raft domains. Given the controversy concerning lipid rafts, it is noteworthy that these approaches may be useful for demonstrating the formation of membrane domains in other organisms, including eukaryotic cells. The use of complementary domain detection techniques involving different principles greatly reduces the possibility that experimental artifacts can explain these results. The possibility of artifacts is also greatly reduced by the use of a large set of sterols to establish a very strong correlation between membrane domain formation and raft forming physical properties, because it tends to rule out alternate explanations based on other variables (e.g. a specific chemical feature found on one particular sterol).

It is important that membrane domain properties in sphingolipid and sterol containing model membranes and those proposed for eukaryotic cells appear to be very similar to those of *B. burgdorferi* domains. Yet *B. burgdorferi* has no sphingolipids. The cholesterol glycolipid ACGal, which has been identified as being enriched in DRM from both *B. burgdorferi* [25] and plant cells [42], is likely to play a role that combines that of sphingolipids and sterols in eukaryotes. However, we cannot rule out some role for proteins or other lipids in *B. burgdorferi*.

### Using FRET to detect ordered domains in *B. burgdorferi*

The FRET protocol devised to detect lipid domain segregation in *B. burgdorferi* may be particularly useful for raft detection in other systems. TMADPH and ODRB are charged hydrophobic probes that readily incorporate into membranes when added externally to cells. Domains larger than Ro for the TMADPH/ODRB pair (∼23 Å calculated as described previously, and assuming all ODRB is membrane bound [38]) can be detected with these probes.

It is unlikely that the fluorescent probes used altered domain formation significantly. In addition to the consistency of FRET results with those obtained with other methods, FRET in untreated cells after halving ODRB concentration showed a temperature dependence similar to that observed at higher ODRB concentrations, and other controls showed that the fluorescent probes did not alter domain formation detected by TEM, cell morphology, or cell growth (not shown).

### Close connection between membrane integrity and ordered domain formation

The parallels between the effects of sterol structure on both cell morphology and membrane integrity were striking. Not only were sterols with the ability to form ordered membrane domains both necessary and sufficient to maintain normal morphology and membrane integrity, but also sterols with an intermediate ability to support ordered membrane domain formation tended to show an intermediate disruption of impermeability and normal morphology. This is consistent with the conclusion that *B. burgdorferi* morphology and membrane integrity require the same sterol properties that support ordered membrane domain formation. Thus, the most obvious interpretation is that the formation of ordered domains is somehow required for membrane integrity. Alternately, these changes could be due to an overall decrease in how tightly outer membrane lipids are packed after sterol substitution. At a minimum, the observation that diverse sterols have effects on membrane integrity that strongly correlate with their physical properties, but not with any specific chemical feature of sterols, tends to rule out alternate hypotheses based on specific chemical features of sterols, as noted above.

The change in membrane morphology and integrity appears to be linked to a change in the normal spirochete flat wave morphology. This raises the question of how these changes are related. It is possible that the loss of the flat wave morphology, which is created by the force exerted by the flagella on the cell wall/inner membrane complex, is due, in turn, to the loss of an external force produced by the outer membrane with ordered domains on the flagella itself, thereby decreasing the flagellar force on the inner membranes. Alternatively, these data may suggest that there is a physical connection between the outer membranes and the flagella. In this interpretation, a putative linking protein between the structures or a cofactor needed for their interaction could be lost when lipid rafts are not formed in the outer membrane. The change in these interactions could compromise flagellar structure. Finally, there could be an interaction between the outer membrane and peptidoglycan or inner membrane that when altered affects flagella. It is even possible that the inner leaflet has rafts perturbed by sterol substitution. Physical manipulations of the specimens are an unlikely explanation of the altered morphology, as all the experiments with the different sterols were done in the same manner and some sterols altered morphology whereas others did not.

Based on this, we explain the release of proteins associated with the change in membrane permeability as due to outer membrane disruptions (resulting in detection of released flagellar subunits) followed shortly by the death of the spirochete (resulting in detection of released cytoplasmic DnaK). We think that the initial changes in the outer membrane eventually render the bacteria sensitive to a secondary osmotic lysis event. Although this behavior by itself is not informative about the mechanism of raft or sterol interactions with other parts of the bacteria, it does tell us that the changes associated with substitutions involving sterols that cannot properly support raft formation can have catastrophic consequences for the bacterium, and therefore are functionally important.

The observation that altering lipid composition lead to deleterious changes in *B. burgdorferi* could have biomedical implications, given the large fraction of antibiotics that act by attacking bacterial membrane integrity. Development of drugs interfering with *B. burgdorferi* cholesterol glycolipid synthesis or sterol uptake may represent a novel therapeutic approach to combat *B. burgdorferi* infections and that of other pathogenic cholesterol-containing bacteria/microorganisms.

In this regard, it is interesting that a similar dependence upon sterol physical properties has been observed for maintenance of membrane integrity after freezing and thawing in yeast cells [43]. Modestly raft-supporting zymosterol increased yeast cell membrane permeability while the strongly raft-forming ergosterol did not [43]. Additionally, model membranes depleted of sterols or containing lanosterol have been shown to have a greater permeability than those containing cholesterol [44].

That domain-forming sterols are necessary to maintain membrane integrity and viability of *B. burgdorferi* is not surprising from an evolutionary standpoint. The life cycle of *Borrelia* involves infection of a tick vector or mammalian host, as these organisms are never free-living [3]. The cells of mammals and many species of ticks contain cholesterol, so it is logical to assume that *B. burgdorferi* has evolved a preference for domain-forming sterols, like cholesterol, as these would be the predominant sterols available during the spirochete life cycle.

## Materials and Methods

### Materials

1, 2- dipalmitoyl-sn-glycero-3-phosphatidylcholine (DPPC), 1-palmitoyl-2-oleoyl-phosphatidylcholine (POPC), 1, 2- dioleoyl-sn-glycero-3-phosphatidylcholine (DOPC), zymosterol, and cholesterol were purchased from Avanti Polar Lipids (Alabaster, AL). Other sterols were purchased from Steraloids Inc. (Newport, RI). Lipids were stored in chloroform or ethanol at −20°C. (Ethanol was the solvent for sterols used for substitution in cells whereas chloroform was solvent for sterols used for the model membrane experiments.) Lipid concentrations were determined by dry weight. The fluorescent probes 1-(4-trimethylammonium)-6-phenyl-1, 3, 5-hexatriene *p*- toluenesulfonate (TMADPH) and octadecyl rhodamine B (ODRB) were purchased from Molecular Probes division of Invitrogen, stored in ethanol at −20°C and concentrations determined by absorbance using ε = 84,800 M^−1^ cm^−1^ at 353 nm and 125,000 M^−1^ cm^−1^ at at 555 nm respectively. 1,6-Diphenyl-1,3,5-hexatriene (DPH) was purchased from Sigma-Aldrich (St Louis, MO) and its concentration determined by absorbance using ε = 84,800 M^−1^ cm^−1^ at 353 nm in ethanol. High performance thin layer chromatography (HP-TLC) plates (Silica Gel 60) were purchased from VWR International (Batavia, IL). HP-TLC analysis of 10 µg samples of sterols chromatographed in 1∶1 ethyl acetate/hexane [28] showed at most minor impurities after long term storage except in the case of stigmasterol, and especially ergosterol. However, ergosterol did not show impurities prior to storage, and anisotropy measurements on sterol-substituted cells confirmed that ergosterol retained its ability to support a high degree of membrane order after addition to cells. Analysis of total *Borrelia* lipids by TLC was performed as described previously [25].

### Bacteria and growth conditions

*Borrelia burgdorferi* strain B31 was used for all studies and grown in BSK-H medium (Sigma) under microaerophillic conditions at 33°C.

### Sterol substitutions

To substitute different sterols for cholesterol in live *B. burgdorferi* for various studies the following procedure was used. Cultures of 50 ml with spirochetes grown to about 1×10^8^ cells/ml were pelleted from the BSK-H culture medium by centrifugation at 5000× g for 10 min at room temperature. The pellet was resuspended in 50 mL Hank's Balanced Salt Solution (HBSS) and washed twice. After the last wash, the pellet was resuspended in 48.5 mL of HBSS to which 2.5 mL of 200 mM MβCD (Invitrogen) in PBS was added for a final concentration of 10 mM MβCD. After incubation for 30 min at 33°C, the spirochetes were centrifuged and resuspended in HBSS. For propidium iodide assays and spirochetes intended for growth-recovery assays the cell concentration was 1×10^8^ spirochetes/mL. For TEM analysis the cell concentration was 5×10^6^ spirochetes/mL. For SDS-PAGE and Western blot analysis of supernatants, FRET analysis of live *B. burgdorferi*, and isolation of DRM from whole *B. burgdorferi* the cell concentration was 4×10^8^ spirochetes/mL.

Once spirochetes were resuspended in HBSS, sterols stored in 100% ethanol (at 2 mg/ml) or an equivalent amount of ethanol were warmed to room temperature and added to the bacteria at a final sterol concentration of 10 µg/ml and incubated at 33°C, generally for 30 min before proceeding to the next step, unless otherwise noted. For studies investigating osmotic lysis of *B. burgdorferi* was investigated, spirochetes were treated in the same manner with the exception of the osmoprotectant, dextran T500 (6% w/v, Pharmacia), being present in the HBSS.

### Propidium iodide assays

*B. burgdorferi* were tested for differences in membrane permeability following sterol substitutions by propidium iodide staining. Sterol-substituted spirochetes at a concentration of 1×10^8^ spirochetes/mL incubated at 33°C in HBSS were stained with propidium iodide for 15 minutes at 33°C using the LIVE/DEAD BacLight Bacterial Viability kit (Invitrogen) according to the manufacturer's directions, at 45 min, 2 h 15 min or 4 h 45 min after .following the initial sterol substitution step. Propidium iodide fluorescence was measured in a SpectraMax M2 plate reader (Molecular Devices) at an excitation of 535 nm and an emission of 617 nm.

### SDS-PAGE and Western blots

Sterol-substituted *B. burgdorferi* were analyzed for protein release, a marker of spirochete death, by SDS-PAGE and Western blot analysis of supernatants from sterol-substituted *B. burgdorferi* at a concentration of 4×10^8^ spirochetes/mL incubated in HBSS at 33°C for 5 hours. Supernatants were run on 12.5% SDS-PAGE gels and either stained with Coomassie blue R-250 for total protein staining or transferred to nitrocellulose for Western blot analysis. Blots were probed with the monoclonal antibodies CB 312 (anti-*B. burgdorferi* DnaK, mouse IgG) or CB1 (anti-*B. burgdorferi* FlaB, mouse IgG) followed by secondary anti-mouse IgG infrared conjugate (IR800, Rockland Immunochemicals). Blots were read in an Odyssey Infrared Scanner (LI-COR) at a wavelength of 800 nm.

### Detergent-resistant membrane isolation from *B. burgdorferi*

Detergent-Resistant Membranes (DRM) were isolated from sterol-substituted *B. burgdorferi* at a concentration of 4×10^8^ spirochetes/mL using the Caveola/Rafts Isolation Kit (Sigma) as previously described [25]. DRM and soluble fractions were analyzed by both slot blot (Hoefer) and ELISA. For slot blots, fractions were loaded onto nitrocellulose and probed with anti-asialo GM1 (for sterol glycolipids, rabbit polyclonal IgG, AbCam) or monoclonal antibodies CB2 (anti-OspB, mouse IgG) or CB10 (anti-OspA, mouse IgG) [45], [46]. Anti-Rabbit IgG IR700 conjugate or anti-mouse IgG IR800 conjugate were used as secondary antibodies. Blots were read in an Odyssey Infrared Scanner (LI-COR) at wavelengths of 700 and 800 nm.

DRM and soluble fractions were also analyzed by ELISA by combining fractions with coating buffer (50 mM Na_2_CO_3_, 50 mM NaHCO_3_, pH = 9.6), to coat the wells of ELISA plates at 4°C overnight. After blocking with 2% BSA, wells were probed with the antibodies mentioned above, except that anti-rabbit IgG or anti-mouse IgG alkaline phosphatase conjugates (Sigma) were used, followed by development with p-nitrophenyl phosphate (pNPP, Sigma) measured at 405 nm in a SpectraMax M2 plate reader.

### Biotin-labeled lipid incorporation into *B. burgdorferi*

The lipid probes biotin-PEG-DPPE and biotin-PEG-DOPE [40] were used to label whole *B. burgdorferi* by adding aliquots from 10 mM ethanol stocks solutions of the probes to live spirochetes in HBSS to a final concentration of 60 µM. After addition of the probes, spirochetes were incubated at 33°C for 1 h.

### Negative-stain immunogold TEM analysis of whole *B. burgdorferi*

Sterol-substituted or biotin lipid probe-labeled *B. burgdorferi* at a concentration of 5×10^6^ spirochetes/mL were adhered to grids, fixed in 1% (v/v) glutaraldehyde, and washed as described previously [41] to prepare organisms for negative-stain TEM analysis, and probed with anti-asialo GM1 (rabbit IgG) plus in some cases anti-biotin (mouse IgG)(AbCam) for 1 hour. Anti-rabbit IgG and anti-mouse IgG colloidal gold conjugates (6 nm and 15 nm, respectively; Jackson Immunochemicals) were used as secondary antibodies. Following labeling with colloidal gold conjugates, grids were stained with PTA and analyzed by TEM as previously described [41].

### TEM image analysis

To quantify the clustering of the cholesterol glycolipids, we analyzed the TEM micrographs using a common linear transformation of Ripley's K-function [47]–[49]

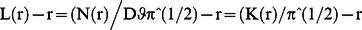
where *N(r)* is the number of particles within a radius *r* of a given particle and *D* is the average particle surface density. When the particles are randomly distributed, *L(r)-r = 0*±*the* confidence interval (CI) for all values of *r*. If the function is positive for any value of r, the particles are clustered on that length scale. The confidence index (CI) was estimated by simulating 100 random particle distributions (on a total surface and with *D* comparable to those of the examined samples) and calculating each time *L(r)-r*. The CI(*r*) was then determined as two times the standard deviation from the average simulated *L(r)-r* curve. To avoid edge effects [47] we analyzed the number of neighbors only for particles that were >80 nm away from the edge of the image of the bacterial cell. Each image measured was chosen at random and covers approximately 400 nm along the length of the spirochete. For each sterol, we analyzed three different micrographs, one from each of three different bacteria, each bacterium being from a different batch and separate sterol substitution experiments.

To quantify the co-localization of cholesterol glycolipids antibodies (linked to “small” gold particles) and biotinylated lipids (linked to “large” gold particles), a slightly different analysis was used. We defined a co-localization parameter *C_40_*. We first counted the number of small gold particles (n_40 nm_) within a radius of 40 nm of each large particle. *C_40_* was then calculated by normalizing n_40_ as a percentage of the total number of small particles (N), i.e C_40_ = n_40 nm_/N×100%. All quantitative image analyses and simulations were performed using self-written algorithms (Matlab, The MathWorks, Natick, MA)

### Lipid extraction from *B. burgdorferi* and thin layer chromatography

Sterol-substituted or sterol-depleted *B. burgdorferi* at a concentration of 4×10^8^ spirochetes/mL were analyzed for lipid composition by extracting lipids after pelleting, using chloroform-methanol (1∶2 v/v) [50]. Lipid extracts were resolved on HPTLC silica plates (EM separations) chromatographed in chloroform-methanol (85∶15) and stained with iodine vapor. Lipid profiles were analyzed using known Rf values from identical solvent systems [9], [10], [25].

### Preparation of model membrane vesicles

Small unilamellar vesicles (SUV) were prepared by ethanol dilution in a manner similar to that described previously [38]. Lipids stored in chloroform at −20°C were warmed to 23°C, pipeted into glass tubes, mixed and dried under N_2_, dissolved in 20 µL ethanol and dispersed in 980 µL 10 mM Na_2_HPO_4_, 1 mM KH_2_PO_4_, 137 mM NaCl and 2.7 mM KCl, pH 7.4 at 70°C. SUV contained 100 µM lipid and were incubated at room temperature for 1 h.

### Fluorescence, absorbance and fluorescence anisotropy measurements

Fluorescence was measured on SPEX Fluorolog 3 spectroflorimeter (Jobin-Yvon, Edison, NJ) using quartz semimicro cuvettes (excitation path length 10 mm and emission path length 4 mm). Slit-width band-widths were set to 4 nm (2 mm physical size) for excitation and emission. TMADPH fluorescence was measured at an excitation λ of 358 nm and emission λ of 430 nm, and values corrected for background fluorescence. Absorbance was measured using a Beckman 640 spectrophotometer (Beckman Instruments, Fullerton, CA) using quartz cuvettes. DPH fluorescence anisotropy measurements were made using SPEX automated Glan-Thompson polarizer accessory with slit-width band-widths set to 4.2 nm (excitation) and 8.4 nm (emission). A 1.25 µL aliquot of ethanolic DPH from a stock solution of 198 µM was added per mL of cells in HBSS (at 10^7^ cells/mL), and incubated for 10 min at 24°C and immediately after stirring samples anisotropy was measured.

### FRET measurement in *B. burgdorferi* and model membranes

Spirochetes or sterol substituted spirochetes at a concentration of 4×10^8^/mL in HBSS were used for the FRET measurements. An aliquot of 5.2 µL (from a 73 µM ethanolic stock solution) of FRET donor TMADPH was added to 4 mL of spirochetes, mixed well by stirring and incubated at room temperature for 10 min. The spriochetes were divided into four 900 µL aliquots, placed in quartz cuvettes and heated to 35°C. Donor fluorescence intensity of all four samples was measured before adding acceptor. Two samples out of the four were defined as F samples, and to them 5.2 µL (out of a 322 µM ethanolic stock solution) of the acceptor, octadecyl rhodamine B (ODRB), was added, followed by incubation at 35°C for 15 min. FRET measurements were then initiated on the F samples and Fo samples (the two samples containing donor but not acceptor). Fluorescence in background samples lacking donor and ones lacking donor but containing acceptor was also measured. The temperature of the samples was measured using a probe thermometer placed in a cuvette. (Fisher brand traceable digital thermometer with a YSI microprobe, Fisher Scientific). The cuvette temperature was slowly decreased from 35°C to 15°C in steps of 5°C and the cells were incubated at each temperature for 7 min once the temperature stabilized. Donor fluorescence intensity of Fo and F samples was measured at each temperature and the ratio of TMADPH fluorescence intensity in the presence of acceptor to its absence (F/Fo) was calculated and plotted as a function of temperature. The average values shown come from bacteria from two independent sterol substitution experiments. Background fluorescence was measured at 35 and 15°C and averaged for the two temperatures because they were found to be independent of temperature. The backgrounds were ≤2% of the TMADPH fluorescence signal and were subtracted from the FRET sample values.

FRET measurement in model membranes was carried out similarly to that in cells with the following changes. FRET donor, 0.1 µM TMADPH was added to SUV prepared by ethanol dilution containing 100 µM lipid and was allowed to incubate at 50°C for 10 min. FRET acceptor, 3 µM ODRB, was added to the F samples and further incubated for 10 min after which the cuvettes were cooled to 45°C and fluorescence measurements were initiated. Cuvettes were further cooled slowly to 15°C in steps of 5°C increments and the fluorescence measured once the temperature stabilized.

## Supporting Information

Text S1 Contains Table S1, Figure S1, Figure S2, Figure S3, Figure S4, Figure S5, Figure S6, Figure S7.
(DOC)
