# Supplementary material for: Proving Lipid Rafts Exist: Membrane Domains in the Prokaryote Borrelia burgdorferi Have the Same Properties as Eukaryotic Lipid Rafts
Source: PLoS Pathog. 2013 May 16;9(5):e1003353. doi: 10.1371/journal.ppat.1003353 (PMC3656094; doi:10.1371/journal.ppat.1003353)
Supplement: Text S1 — Contains Table S1, Figure S1, Figure S2, Figure S3, Figure S4, Figure S5, Figure S6, Figure S7. (DOC) [file ppat.1003353.s001.doc]

**Text S1. Supporting Material for LaRocca et al. “Proving Lipid Rafts Exist: Membrane Domains in the Prokaryote *Borrelia burgdorferi* Have the Same Properties as Eukaryotic Lipid Rafts”**

Table S1. Sterols used for sterol-substitution experiments in *B. burgdorferi* and their ability to form lipid raft domains in model membranes are shown.

| **Sterol** | **Domain-forming ability**  **In model membranes** |
| --- | --- |
| Ergosterol | Strong |
| Cholesterol | Strong |
| Dihydrocholesterol | Strong |
| Stigmasterol | Strong |
| Desmosterol | Intermediate |
| Lanosterol | Intermediate |
| Zymosterol | Intermediate |
| Cholesterol Formate | Intermediate |
| Coprostanol | Inhibitory |
| Androstenol | Inhibitory |

Figure S1: Effect of substitution with different sterols upon *B. burgdorferi* lipids. Thin layer chromatography (chloroform:methanol; 85:15; stained with iodine vapor) of lipid extracts from *B. burgdorferi* that have undergone sterol substitutions or have been depleted of sterols. Sterol-depletion results in a disappearance of ACGal, and CGal and free sterol (*). Most sterol substitutions result in the reappearance of lipids with the mobility of ACGal, and CGal and free sterol. It is not clear if this represents incorporation of the new sterol or residual free cholesterol into these lipids. Sterol substitution with ergosterol resulted in the reappearance of free sterol and a lipid with the mobility of ACGal but not CGal (*). In the case of coprostanol and androstenol the lack of a spot with the mobility of free sterol may reflect altered TLC mobility of these sterols. U = untreated, depl = sterol-depleted, ergo = ergosterol, chol = cholesterol, lano = lanosterol, zymo = zymosterol, copr = coprostanol, andr = androstenol. Several additional experiments in which the sum of sterol glycolipid levels were evaluated on Western-type blots of gels using anti-asialo GM1 showed a pattern for total sterol glycolipid levels vs. sterol substitution similar to that shown below.

Figure S2. Detection of membrane order in control *B.burgdorferi* cells and *B.burgdorferi* with substituted sterols via measurement. DPH fluorescence anisotropy was used to measure membrane order at 24^o^C. The mean and standard deviation for four experiments is shown. Notice that the order (anisotropy) is strongly correlated with sterol raft-forming ability (see Supplemental Table 1).

Figure S3. Representative negative-stain TEM images of *B. burgdorferi* substituted with the indicated sterols and probed for sterol glycolipids as in Figure 1. TEM after sterol substitution is shown for sterols not shown in Figure 1. Size bars = 100 nm.

Figure S4. Ripley analysis values at A. r = 30 nm, and B. r =60 nm, from the data in Figure 1B. Error bars represent standard deviations from three separate
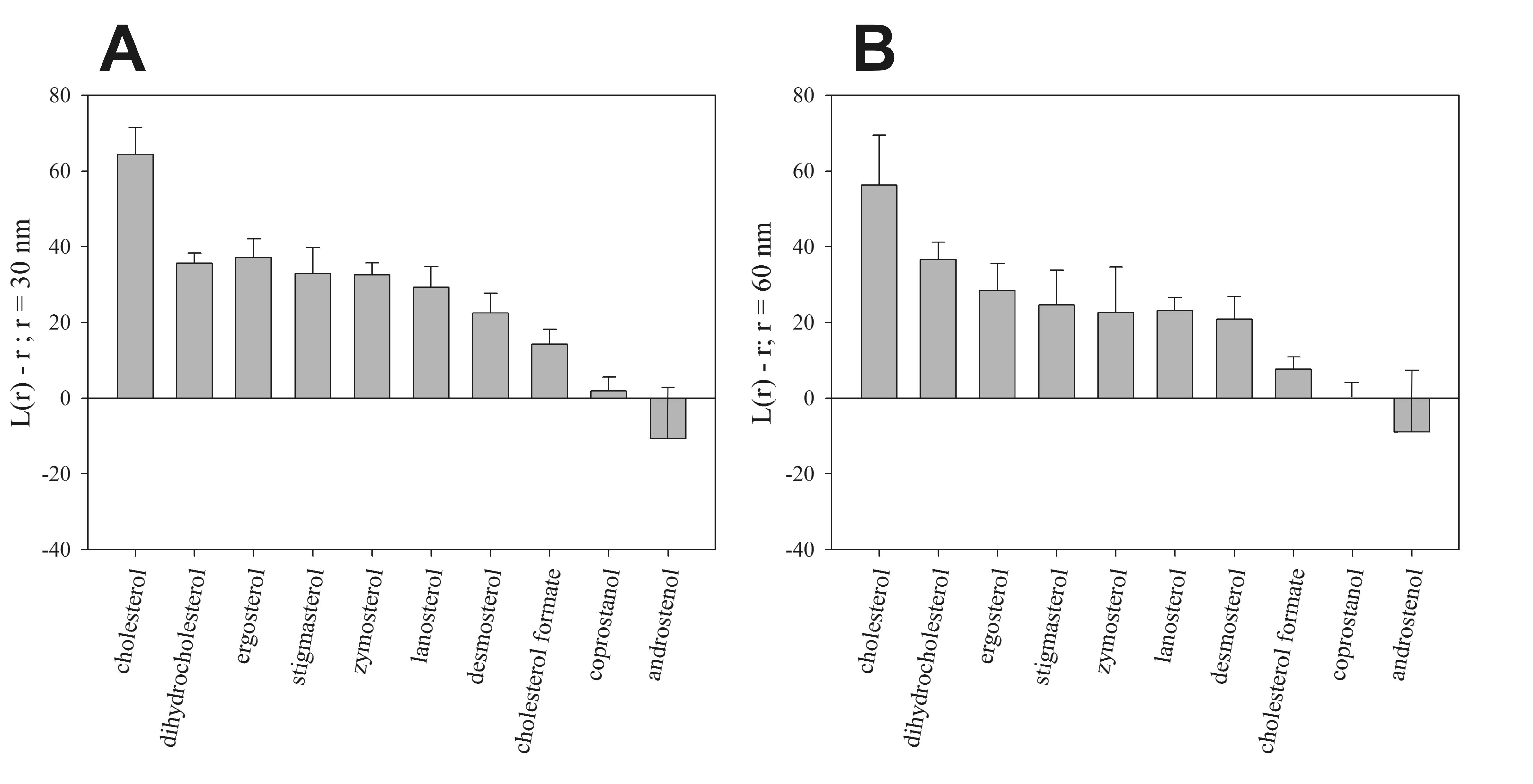
experiments.

Figure S5. Dependence of FRET-detected domain formation in *B. burgdorferi* upon sterol composition. Samples are those from Figure 2. F/Fo is the ratio of TMADPH fluorescence in the presence of ODRB to that in its absence. Δ F/Fo is the difference between F/Fo at 35^o^C and that at 15^o^C. Temperature dependent domain formation is detected by a positive Δ F/Fo value. The experimental conditions are: chol = cholesterol substituted cells, ergo = ergosterol substituted cells, dihydro = dihydrocholesterol substituted cells, untreat = untreated cells, deplet = cholesterol lipid depleted cells, lano = lanosterol substituted cells, zymo = zymosterol substituted cells, andro = androstenol substituted cells, copro = coprostanol substituted cells. Mean and standard deviation from four samples is shown, except for the untreated and lanosterol samples for which the average and range of duplicates is shown.

Figure S6**:** Sterol-dependence of OspA and OspB association with ordered domains in *B. burgdorferi* as determined by detergent resistance. Levels of A. and C. OspB, or B. and D. OspA in DRM (A. and B.) or soluble fractions (C. and D) are shown. The levels of lipid raft-associated OspB, and OspA are highest in DRM fractions from *B. burgdorferi* substituted with ergosterol, and cholesterol, intermediate values for substitution with lanosterol or zymosterol, and lowest values for substitution with coprostanol or androstenol or sterol-depleted. Mean and SEM from three experiments is shown. One-way ANOVA, *** P < 0.001, ** P < 0.01.

Figure S7 Representative immunoblots showing partitioning of cholesterol glycolipids (anti-asialo GM1) and two outer membrane lipoproteins (OspB and OspA) in DRM fractions following TX-100 treatment at 33^o^C and separation on a density gradient. The cytosolic control, Lon-1 protease, partitions into the soluble fraction, as expected. Percentages refer to percent of OptiPrep in the density gradient solution. Osp proteins are lost in the 20-30% fractions when lipid rafts are destroyed by sterol depletion .
